# Response to Comments by Vuksan V. et al., *Nutrients* 2017, *9*, 398, Regarding an Article by Solah V.A. et al., *Nutrients* 2017, *9*, 149

**DOI:** 10.3390/nu9040408

**Published:** 2017-04-20

**Authors:** Vicky A. Solah, Deborah A. Kerr, Wendy J. Hunt, Stuart K. Johnson, Carol J. Boushey, Edward J. Delp, Xingqiong Meng, Roland J. Gahler, Anthony P. James, Aqif S. Mukhtar, Haelee K. Fenton, Simon Wood

**Affiliations:** 1 School of Public Health, Faculty of Health Sciences, Curtin University, Perth, WA 6845, Australia; d.kerr@curtin.edu.au (D.A.K.); w.hunt@curtin.edu.au (W.J.H.); s.johnson@curtin.edu.au (S.K.J.); T.P.James@curtin.edu.au (A.P.J.); Aqif.Mukhtar@curtin.edu.au (A.S.M.); h.fenton@curtin.edu.au (H.K.F.); simonwood@shaw.ca (S.W.); 2 Epidemiology Program, University of Hawaii Cancer Center, Honolulu, HI 96813, USA; cjboushey@cc.hawaii.edu; 3 Video and Image Processing Laboratory, School of Electrical and Computer Engineering, Purdue University, West Lafayette, IN 47907, USA; ace@ecn.purdue.edu; 4 Flinders Centre for Innovation in Cancer, School of Medicine, Flinders University, Adelaide, SA 5001, Australia; rosie.meng@flinders.edu.au; 5 Factors Group R & D, Burnaby, BC V3N4S9, Canada; rgahler@naturalfactors.com; 6 Curtin Health Innovation Research Institute, Faculty of Health Sciences, Curtin University, Perth, WA 6845, Australia; 7 Centre for Population Health Research, Faculty of Health Sciences, Curtin University, Perth, WA 6845, Australia; 8 InovoBiologic Inc., Calgary, AB Y2N4Y7, Canada; 9 Food, Nutrition and Health Program, University of British Columbia, Vancouver, BC V6T1Z4, Canada

To the Editor:

We have read the comments by Dr. Vuksan regarding our article entitled “Effect of Fibre Supplementation on Body Weight and Composition, Frequency of Eating and Dietary Choice in Overweight Individuals” as published in *Nutrients* in February 2017 [1].

The objective of the research was to determine the effectiveness of fibre supplementation with the viscous and gel-forming fibre, PolyGlycopleX (PGX), on body weight and composition and to determine if frequency of eating and diet can explain subsequent changes in overweight adults.

We are grateful to Vuksan et al. for alerting us to the errors in the manuscript. The labelling of our ‘per-protocol analysis’ as ‘intention to treat’ in Table 4 was an inadvertent error.

In addition, the labelling of Table 5, ‘Change in participant characteristics from baseline to week 12 of the PGXS, PGXG and RF interventions in the subgroup analysis of those who consumed the recommended dose of fibre supplements’ was correct but the words ‘per-protocol analysis’ in brackets was an inadvertent error and has since been removed.

These errors were carried over to the sections that describe the results. The headings of Sections 3.1.1, 3.2.1 and 3.3.1 should be per-protocol analysis and the headings of Sections 3.1.2, 3.2.2 and 3.3.2 should be subgroup analysis.

Other than the errors stated above, the results of the research study are valid. We apologise for these errors and have requested *Nutrients* to publish an Erratum.

In Table 5, the serves of fibre are correct as stated, with 1 serve equal to 3.8–4.4 g (Table 1), so the minimum 2.5 serves per day equals 9.5 g fibre for PGXS and 11 g of fibre for PGXG. The weight of one serving size is 4.5–5 g; for example, 5 g PGXG contains 4.4 g fibre (see Table 1).

Our study is a double-blind research study. The PGX granules, rice flour and PGX softgel were blinded to participants and, although the softgels were in a different form, participants were blind to the content of the capsule. Research staff were blinded to the treatment allocation until all analyses were completed. Therefore no research staff dealing with participants knew if softgels contained the fibre or rice flour.

The purpose of the clinical trial registration was to register our plan for the research and intentions at the time. Further analyses of the data including biochemistry measures is in progress and we will update the trial registry on completion.

We declared the following in Acknowledgments:

PGX^®^ and PolyGlycopleX^®^ are registered trademarks of InovoBiologic Inc., Calgary, AB, Canada. Financial support for the submitted work was provided by Factors Group, Australia Pty. Ltd. RJG owns the Factors Group of Companies, which retains an interest in PGX. SW receives consulting fees from InovoBiologic Inc.

This information could have been placed in the ‘Conflict of Interest’ section of the paper but we still believe there is no conflict of interest as RJG and SW were not involved in data collection or analysis of the results. The study was conducted in Australia and RJG and SW work and live in Canada.

The paper is in full compliance with *Nutrients* Instructions for Authors and Publication Ethics Statement. The authors plainly disclosed in the paper prior to submission the source of financial support for the submitted work, the relationship between RJG and The Factors Group of Companies and the relationship between SW and InovoBiologic Inc. It is unclear how issued patents and published patent applications, some filed as early as 2006, are relevant to the present paper and to any potential conflicts of interest. Further, this type of patent information is publicly available.

We stand by our conclusions that the results demonstrate the potential benefit for the PGX fibre in controlling frequency of eating and in weight loss.
